# Evaluation of a Novel Suture-Button Technique for Open Reduction and Internal Fixation in Isolated Fractures of the Greater Tuberosity of the Humerus: A Retrospective Study

**DOI:** 10.7759/cureus.81514

**Published:** 2025-03-31

**Authors:** Christos Koukos, Dimitrios Giotis, Michael Troullinakis, Dimitrios Karadimos, Paolo Arrigoni, Emilios E Pakos, Christos Lyrtzis, Alexandros Tsitsikas, Fredy Montoya

**Affiliations:** 1 Orthopaedics, Sports Trauma and Pain Institute, Thessaloniki, GRC; 2 Orthopaedics, General Hospital of Ioannina "G. Hatzikosta", Ioannina, GRC; 3 Orthopaedics and Trauma Surgery, Gelenk- und Sportklinik Rhein/Ruhr, Mülheim, DEU; 4 Orthopaedics and Traumatology, ASST Gaetano Pini - CTO, Milan, ITA; 5 Orthopaedics and Traumatology, University Hospital of Ioannina, Ioannina, GRC; 6 Anatomy, Aristotle University of Thessaloniki, Thessaloniki, GRC; 7 Orthopaedics, Clínica Sanatorio Aleman, Universidad de Concepción, Concepción, CHL

**Keywords:** greater tuberosity, humeral head fracture, rotator cuff, suture button, zip tight

## Abstract

The purpose of the study is to present a novel technique for the open reduction and fixation of isolated displaced greater tuberosity (GT) fractures, utilizing a suture-button system, along with an evaluation of its efficacy. We conducted a retrospective study, including 18 patients with isolated tuberosity fractures displaced by more than 5 mm. All patients underwent surgical treatment using open reduction and internal fixation with a suture-button system (Zip Tight), reinforced with high-resistance sutures. The "Constant score," the "ASES (American Shoulder and Elbow Surgeons) score," and range of motion (ROM) were assessed postoperatively, with a minimum follow-up of 18 months. Postoperative X-rays were obtained in the anteroposterior (AP) view with the humerus in a neutral position, as well as in internal and external rotation and axial views. The "Constant score," the "ASES score," and ROM increased significantly, reaching their highest levels at the six-month follow-up and remaining stable up to the 12- and 18-month follow-ups. Postoperatively, the mean anterior flexion reached 145° (range: 100°-170°), and the mean abduction was 142° (range: 95°-170°). At 12 months, no patients exhibited significant problems with internal rotation, with the average reaching the T12 level (range: T7 to the posterior iliac crest). External rotation results were also very satisfactory, with a mean range of 75° (range: 60°-90°). Conclusively, this novel technique could provide a safe and effective surgical solution for the treatment of isolated GT fractures, with minimal complications and positive functional and radiological outcomes.

## Introduction

Proximal humeral fractures account for approximately 5% of all fractures, with up to 20% involving isolated fractures of the greater tuberosity (GT) [1-3]. Although the exact mechanism of injury remains unclear, the initial theory that these fractures result solely from a bony avulsion of the rotator cuff does not consistently apply [4]. The literature generally supports surgical treatment for isolated GT fractures in healthy patients exhibiting more than 5 mm of superior displacement [3,5,6]. However, some authors advocate lowering this threshold to more than 3 mm, citing benefits from immediate surgical reduction and fixation [7-9].

Numerous techniques have been proposed for the osteosynthesis of GT fractures, with fracture morphology serving as the most significant criterion for selecting the appropriate surgical approach. Both arthroscopic and open surgical techniques have been described, with the choice influenced by factors such as fracture morphology, displacement, location, associated intra-articular injuries, and surgeon preference [6-8,10-15].

Despite several comprehensive systematic reviews on the management of isolated GT fractures, no “gold standard” or universally accepted treatment algorithm has yet emerged [15,16]. Furthermore, the influence of different techniques on clinical outcomes remains unclear due to the paucity of studies directly comparing them [15]. The primary aim of this study is to describe a novel method for the fixation of isolated displaced GT fractures using a suture-button system and to evaluate its efficacy.

## Materials and methods

Study design

A retrospective analysis was performed on data from 18 patients (10 male and 8 female) who underwent open reduction and internal fixation of the GT following an acute fracture. The mean age of the patients was 49 years (range: 23-81). In eight patients, the injury involved the dominant shoulder, and 33.3% of cases were associated with a shoulder dislocation. One patient had a comminuted fracture; seven patients had split fractures, and the remaining cases were avulsion fractures. No depression fractures were observed. One case required revision surgery due to secondary dislocation after primary fixation with two cannulated screws.

Patients with associated labral tears or glenoid fractures requiring surgical intervention were excluded. Additional exclusion criteria included multidirectional instability, skeletal instability, open wounds or nerve damage, active infections, and medical unfitness. All procedures were performed by the same surgeon in a sports injury department at a district hospital. Eligibility for surgery was determined based on admission X-rays showing GT displacement of more than 5 mm.

**Figure 1:**
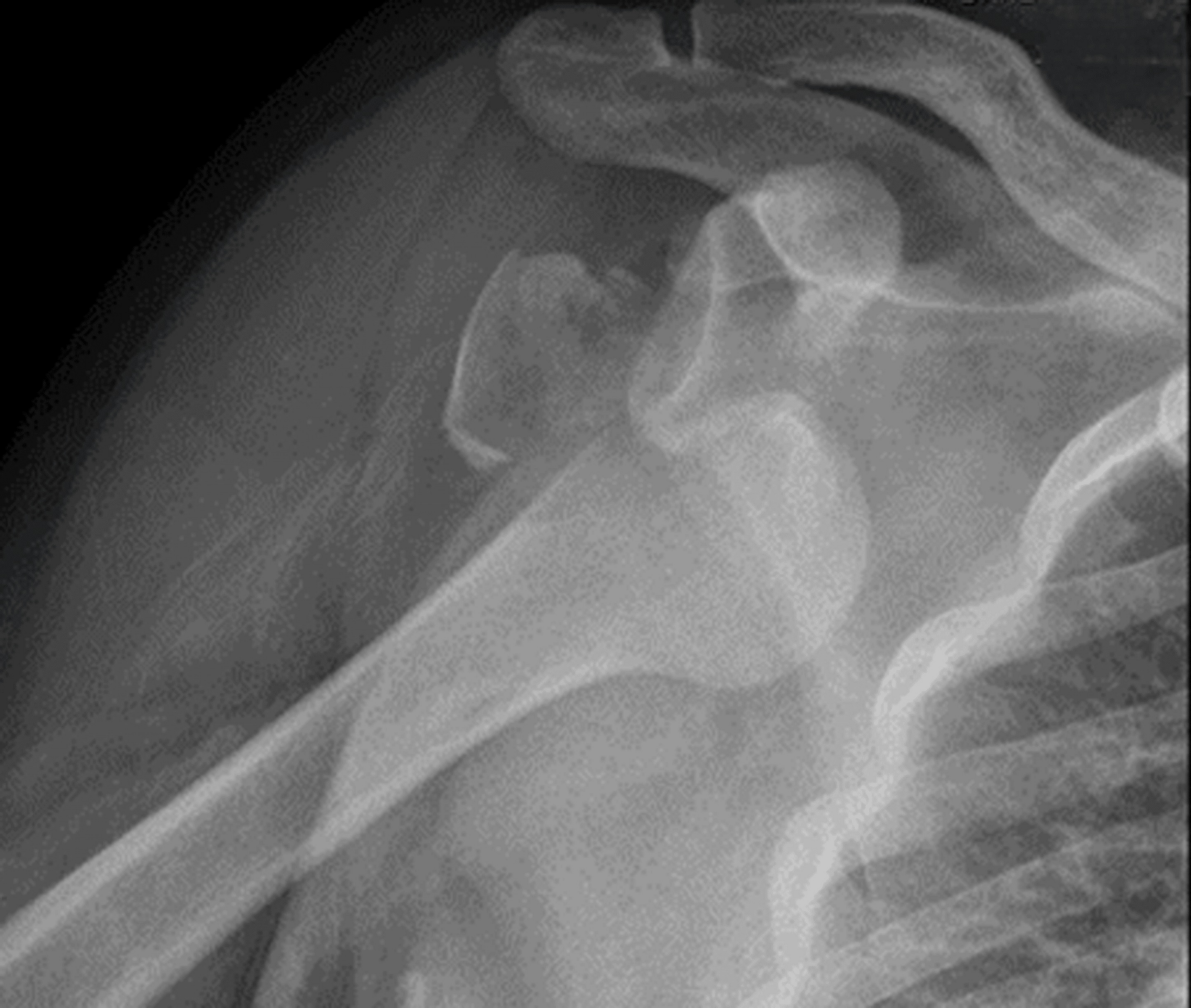
Isolated avulsion fracture of the greater tuberosity following an anteroinferior dislocation of the glenohumeral joint.

Preoperative assessments included radiographs, CT scans, and clinical examinations to confirm the fracture (Figure 1). Due to the acute clinical presentation and significant posttraumatic pain, detailed assessments of shoulder range of motion (ROM) and preoperative scoring were not performed. Follow-up visits were conducted by the surgeon and two fellows, with a minimum follow-up duration of 18 months (range: 18-54 months). Radiological and clinical outcomes were assessed at two weeks, six weeks, three months, six months, and at key clinical benchmarks of 12 months, 18 months, and annually thereafter.

Surgical technique

Under general anesthesia and in the beach chair position, the patient is first inspected. The operative arm is positioned at the edge of the table to allow maximum manipulation and unobstructed fluoroscopic imaging. The acromion, clavicle, and coracoid processes are identified and marked. A standard arthroscopic superior posterolateral portal is then created, and a diagnostic arthroscopy of the glenohumeral joint and subacromial space is performed to rule out any associated labral tears or glenoid fractures requiring intervention.

Next, a standard lateral (deltoid-splitting) approach is used. An approximately 5 cm incision is made, starting from the tip of the acromion and extending distally along the arm. While the incision is typically made at the posterior edge of the clavicle, it can be adjusted according to the specific pathology and fracture location. After superficial dissection of the subcutaneous tissue, the deltoid is split in line with its fibers, no more than 5-6 cm distal to the lateral edge of the acromion, to protect the axillary nerve. A stay suture is placed at the inferior apex of the split to prevent further propagation.

Deep dissection follows, including the excision of the subacromial bursa, which lies directly beneath the deltoid muscle, in order to expose the underlying rotator cuff insertion and proximal humerus. For the superficial dissection, a Kolbel soft tissue retractor or Gelpi retractor is utilized to ensure adequate visibility and working space. Once the deep layers are exposed, a Kolbel self-retaining retractor or Kolbel self-retaining glenoid retractor is used to retract the deltoid muscle fibers, thereby facilitating the manipulation of the underlying tissue.

**Figure 2:**
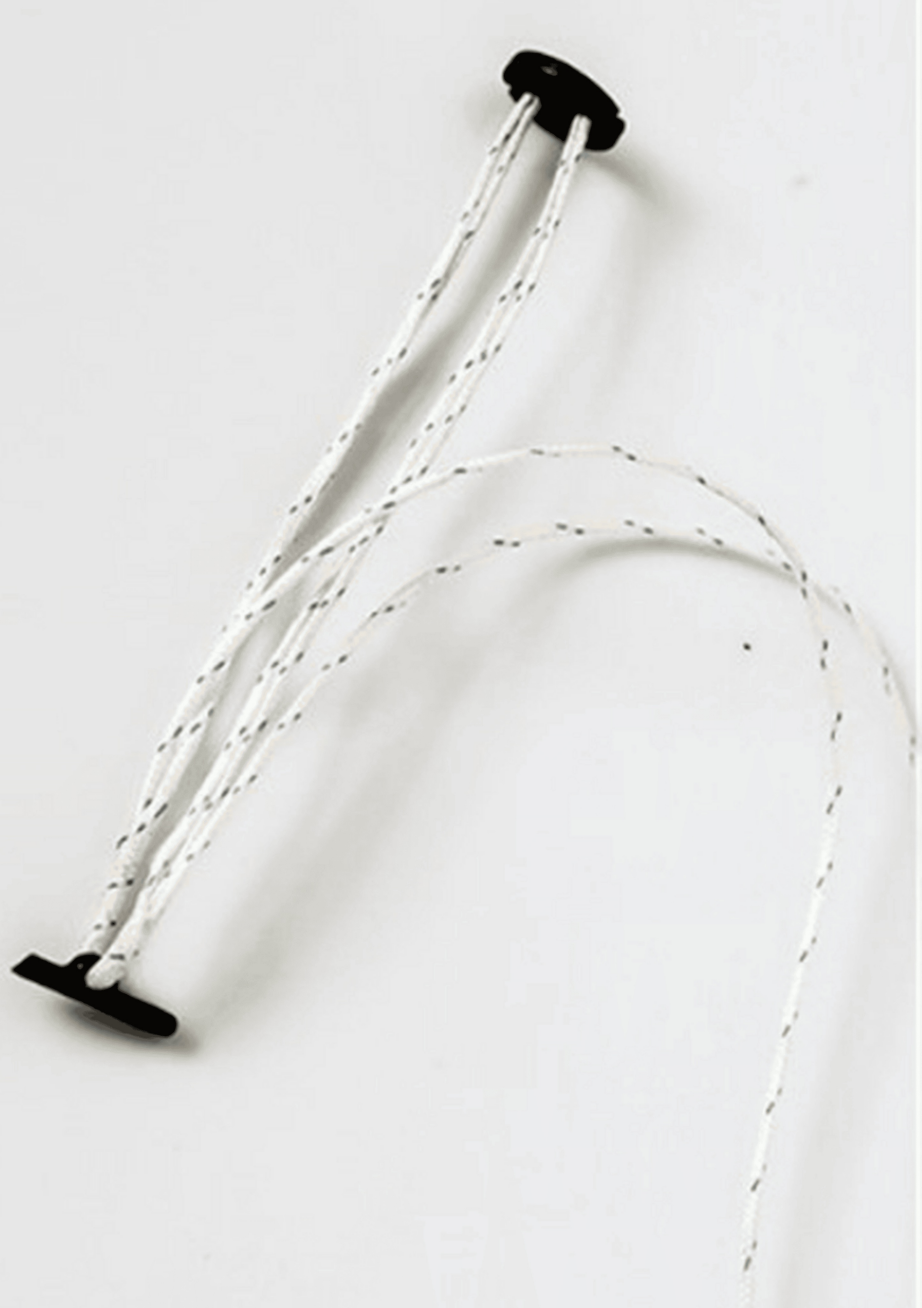
Zip-Tight Fixation System.

The displaced fracture is identified, and after debridement of any smaller fragments or interfering soft tissue remnants, open reduction and preliminary fixation are performed using one or two 1.8 mm K-wires under fluoroscopic guidance. Once anatomical reduction is achieved, a 2.4 mm guide pin is power-drilled through the tuberosity, targeting the calcar under fluoroscopy. The guide pin’s position is verified and adjusted if necessary, and once the correct placement is confirmed, it is left in place. A 4.5 mm ToggleLoc Device Reamer (Zimmer Biomet, Warsaw, IN, USA) is then carefully advanced over the guide pin through the tuberosity and calcar. Caution is taken to avoid advancing medially, to prevent injury to the medial neurovascular structures. Next, the ToggleLoc Fixation Device is inserted using the ToggleLoc pusher from the top. The device is then advanced through the tunnel and deployed on the medial surface of the calcar of the humeral head under indirect fluoroscopic visualization. Subsequently, the ZipLoop (Zimmer Biomet) and its associated zip strand are passed through the tunnel on the tuberosity side and prepared for placement through the round button (Figure 2).

**Figure 3:**
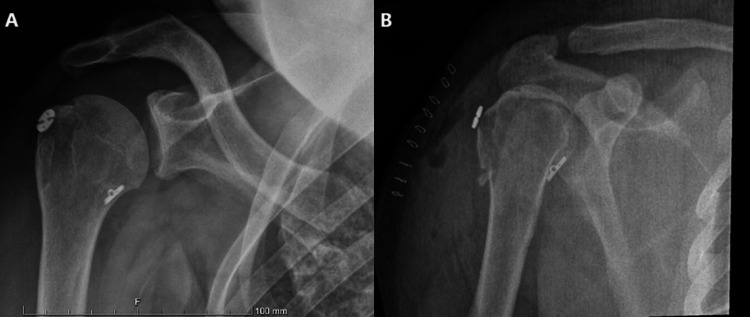
(A)-(B) Humeral head after reduction of the greater tuberosity fracture and fixation with Zip-Tight Fixation System.

The zip strand of the ToggleLoc Device is looped onto the round button, and the blue back-tensioning strand is pulled simultaneously with the zip strand to tighten both buttons together using ZipLoop Technology. While maintaining the reduction of the fracture under direct visualization, additional compression is applied by zipping the buttons together, which is also verified fluoroscopically. Once adequate reduction and compression are achieved, the sutures and blue back-tensioning strands are cut and removed (Figure 3).

**Figure 4:**
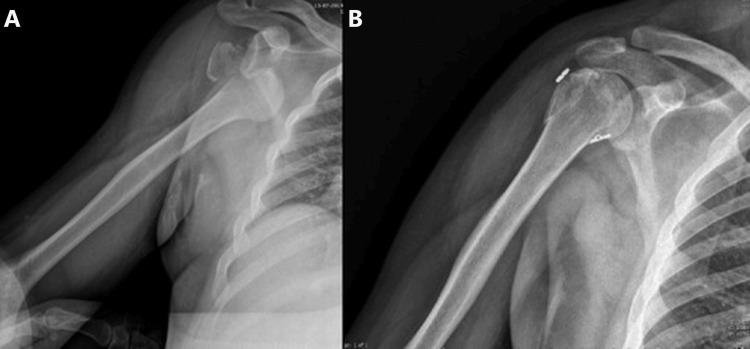
(A) Patient with an isolated fracture of the greater tuberosity following an anteroinferior dislocation of the glenohumeral joint; (B) Postoperative image of the same patient.

Following this, the rotator cuff is re-evaluated for any associated lesions. If necessary, transosseous or side-to-side suturing is performed. Finally, the wound is closed. The deltoid fibers are re-approximated using an absorbable suture in a continuous locking technique. After closing the subcutaneous tissue, the skin is closed with a 3.0 nylon suture, and a shoulder splint is applied for comfort during the first two weeks (Figure 4).

Rehabilitation

Passive motion and rehabilitation are permitted from day 1, allowing up to 90° of abduction and 90° of anterior flexion, with no limitations in passive external rotation or adduction for the first six weeks. Active-assisted abduction and flexion exercises are encouraged, starting at four weeks. Full extension and abduction are allowed six weeks postoperatively, followed by a gradual return to full ROM and muscular strength through isometric and isotonic exercises. Full weight-bearing is permitted after 12 weeks.

Postoperative evaluation

The “Constant score,” the “ASES (American Shoulder and Elbow Surgeons) score,” and ROM were assessed postoperatively [17,18]. Regular X-rays were obtained at defined intervals, with a minimum follow-up of 18 months. Imaging included an anteroposterior (AP) view with the humerus in a neutral position, as well as views in internal and external rotation, and an axial view.

Ethical considerations

The current study was reviewed and approved by the Institutional Review Board of our hospital. Data were collected from all patients who agreed to participate by signing informed consent forms in accordance with the Helsinki Declaration of Principles of 1964, as revised in 2008.

Statistics

The statistical analysis was performed using IBM SPSS Statistics for Windows, Version 26 (Released 2019; IBM Corp., Armonk, NY, USA). Descriptive statistics, including means, ranges, and standard deviations, were calculated for continuous variables. The primary outcome measures, “Constant score,” “ASES score,” and ROM, were assessed at predefined postoperative time points. Paired t-tests were used to compare postoperative scores, with the significance level set at p < 0.05. Repeated measures analysis of variance (ANOVA) was employed to evaluate changes in outcomes over time and to assess the stability of improvements at follow-ups.

## Results

This study included 18 patients, with a median follow-up period of 33 months, and no participants lost to follow-up. The predominant cause and mechanism of injury was a fall on the ipsilateral side, occurring either through a direct impact or an indirect mechanism involving the arm in anterior flexion and abduction. None of the patients had a nerve injury prior to surgery. All patients underwent surgical treatment within the first 10 days after injury.

Postoperatively, the “Constant score” and the “ASES score” gradually increased significantly, reaching their highest values at the six-month follow-up (90, range: 75-100, and 89, range: 73-100, respectively), along with improvements in ROM. These parameters remained stable through the 12- and 18-month follow-ups. At the six-month postoperatively, the mean anterior flexion was 145° (range: 100°-170°), and the mean abduction was 142° (range: 95°-170°). At 12 months postoperatively, no patients experienced significant limitations in internal rotation, with the average reaching the T12 level (range: T7 to the posterior iliac crest). External rotation results were also highly satisfactory, with a mean range of 75° (range: 60°-90°). The mean time for radiological consolidation was 11 weeks (range: 8-13 weeks) (Table 1).

**Table 1:** Functional results at 6, 12 and 18 months postoperatively (post-op). t: t-value; p: p-value; F: F-value; ASES: American Shoulder and Elbow Surgeons None of the comparisons showed statistically significant differences (p > 0.05 for all tests). The paired t-tests suggest no significant difference between any two individual time points. The analysis of variance (ANOVA) test also indicates that there was no significant overall difference across the three time points. Internal rotation was consistently measured as "T12 (T7 sacrum)" at all three postoperative time points for all patients. Since there is no variability in the data across these time points, conducting paired t-tests or ANOVA is not applicable.

	6-month post-op	12-month post-op	18-month post-op	Paired t-test 6-month vs. 12-month post-op	Paired t-test 12-month vs. 18-month post-op	Paired t-test 6-month vs. 18-month post-op	ANOVA (all three time points)
Flexion	145° (100º-170°)	145° (95º-170°)	145° (98º-170°)	t = 1.840 / p = 0.083	t = -0.626 / p = 0.539	t = 0.962 / p = 0.349	F = 0.001 / p = 0.999
Abduction	142° (95º-170°)	141° (90º-170°)	142° (93º-170°)	t = 1.771 / p = 0.094	t = -1.645 / p = 0.118	t = 0.858 / p = 0.402	F = 0.001 / p = 0.999
External rotation	75° (60°-90°)	75° (60°-90°)	74° (55°-90°)	t = 0.980 / p = 0.340	t = 1.369 / p = 0.188	t = 1.414 / p = 0.175	F = 0.008 / p = 0.992
Internal rotation	T12 (T7 sacrum)	T12 (T7 sacrum)	T12 (T7 sacrum)	Not applicable	Not applicable	Not applicable	Not applicable
Constant score	90 (75-100)	90 (73-100)	90 (74-100)	t = 0.385 / p = 0.705	t = 0.362 / p = 0.721	t = 0.255 / p = 0.801	F = 0.002 / p = 0.998
ASES score	89 (73-100)	89 (72-100)	89 (73-100)	t = 0.245 / p = 0.809	t = 0.188 / p = 0.853	t = 0.140 / p = 0.890	F = 0.001 / p = 0.999

At 18 months postoperatively, no clinical or ultrasound evidence of rotator cuff lesions was observed in any patient. Moreover, there were no instances of implant failure or dislocation. Beyond 12 months, no significant changes were noted in any of the scores, and all patients reported stable elbow function at their last assessment.

## Discussion

There is a general consensus in the literature that surgical intervention for isolated GT fractures in healthy patients is warranted when the superior displacement exceeds 5 mm [3,5,6]. This recommendation is largely based on the functional demands of younger, active patients and the anatomical position of the GT beneath the acromion, which may exacerbate biomechanical challenges [7,8].

Platzer et al. suggested that even 3 mm of displacement can alter rotator cuff biomechanics. However, these findings should be interpreted with caution, as their results did not reach statistical significance [7,19]. Until higher-quality studies are available, a threshold of >5 mm of displacement should remain the standard indication for surgical management of GT fractures [19]. Additionally, late migration of the GT fragment may occur in cases with displacement of less than 5 mm [20].

The two major classification systems for proximal humerus fractures are the Neer and AO classifications [10,21]. While the Neer classification does not specifically address GT fractures, the AO classification categorizes them as non-displaced, displaced, or associated with shoulder dislocation, with displacement defined as >5 mm of GT fragment translation from its anatomical position [21]. However, neither classification system has demonstrated good interobserver or intraobserver reliability for assessing proximal humerus fractures [19,22-26].

Although conventional radiography is often criticized for its inability to precisely measure GT displacement, studies suggest that advanced imaging techniques, such as CT scans, have not resolved this issue [22,23,26-28]. Mutch et al. proposed a more specific morphological classification, dividing GT fractures into three types: avulsion, split, and depression. This classification has demonstrated acceptable interobserver and intraobserver reliability [9].

In our study, we present a novel surgical technique for treating displaced isolated GT fractures. Significant clinical improvement was observed as early as the first postoperative follow-up, with all patients reporting very good to excellent shoulder function sustained through to the final follow-up. A potential disadvantage of this technique is the risk of injuring the anterior circumflex vessels on the medial/caudal side of the calcar, or even more medial structures, like the axillary nerve. This risk may arise if the reamer or ToggleLoc pusher is advanced too far medially or caudally, or if the medial button drags and entraps the underlying soft tissue when pulled back onto the calcar.

Another challenge is hardware removal, particularly of the medial button. The deltoid-splitting approach provides limited visualization of medial structures, even with maximal external rotation of the arm. An alternative could be the anterolateral shoulder approach or the Mackenzie approach [29], which offers greater medial exposure. However, these approaches still carry the risk of damaging or sacrificing the circumflex vessels during the extraction of the medial button, when attempting to access the calcar area [29].

Our novel open fixation technique can also be adapted for arthroscopic application. An arthroscopic version could offer benefits such as reduced soft tissue trauma, lower risks of postoperative infections and adhesions, decreased intraoperative blood loss, improved visualization of GT fragments, enhanced detection of accompanying soft tissue lesions, and superior cosmetic outcomes [14,15,30]. However, open fixation provides advantages, including lower cost, a shorter learning curve, and broader applicability across various fracture types [15].

Additionally, our technique can be combined with rotator cuff repair if needed, either during the primary procedure or as a secondary intervention later. The tunnel drilled is only 4.5 mm in diameter, allowing flexibility in choosing between different open or arthroscopic methods based on the tear pattern, morphology, and location. This avoids major concerns about available “real estate” for anchor placement or tunnel conversion, which can be problematic when using plating systems for primary fracture fixation.

Unlike most arthroscopic techniques, our method does not depend heavily on the location of the fracture line in split or avulsion GT fractures, thereby eliminating concerns about fractures being too medial, lateral, or distal, which could be a limitation for suture-anchor techniques [11,12]. Moreover, the cortical fixation of the buttons in our technique reduces the influence of post-traumatic or general cancellous bone quality of the humeral head - a critical factor in suture-anchor arthroscopic procedures.

Compared to plate fixation [13], the low profile of the lateral button minimizes the risk of hardware impingement on the acromion while providing comparable compression values. In vitro biomechanical studies or further clinical trials comparing these techniques could offer more precise insights into these issues. In general, our method is simpler, arguably faster, and reduces the risk of hardware irritation during motion due to the low profile of the inserted button.

Our research has several limitations inherent to its retrospective design. A priori power analysis was not performed. Furthermore, the small number of patients, the lack of a control group, and the absence of long-term follow-up exceeding two years limit the reliability of our conclusions. Another limitation is that the surgeon and the fellows examining the patients during follow-up were not blinded to the procedure.

## Conclusions

In conclusion, our study demonstrates that the suture-button fixation technique can be performed safely and effectively using this fluoroscopic-assisted novel approach in a select group of patients, yielding excellent compression results and satisfactory radiological and functional outcomes. Despite the small number of cases and medium-term follow-up, we believe this novel surgical procedure is a reliable and reproducible option; however, larger studies are needed to validate these findings.
